# Evolution of Treatment in Advanced Cholangiocarcinoma: Old and New towards Precision Oncology

**DOI:** 10.3390/ijms232315124

**Published:** 2022-12-01

**Authors:** Maurizio Capuozzo, Mariachiara Santorsola, Loris Landi, Vincenza Granata, Francesco Perri, Venere Celotto, Oreste Gualillo, Guglielmo Nasti, Alessandro Ottaiano

**Affiliations:** 1 Coordinamento Farmaceutico, ASL-Naples-3, 80056 Ercolano, Italy; 2 Istituto Nazionale Tumori di Napoli, IRCCS “G. Pascale”, Via M. Semmola, 80131 Naples, Italy; 3 Sanitary District, Ds. 58 ASL-Naples-3, 80056 Ercolano, Italy; 4 SERGAS (Servizo Galego de Saude) and IDIS (Instituto de Investigación Sanitaria de Santiago), NEIRID Lab (Neuroendocrine Interactions in Rheumatology and Inflammatory Diseases), Research Laboratory 9, Santiago University Clinical Hospital, 15706 Santiago de Compostela, Spain

**Keywords:** cholangiocarcinoma, ivosidenib, pemigatinib, target therapy, genetics, precision oncology

## Abstract

Cholangiocarcinoma (CCA) is a malignant neoplasm arising in the epithelium of the biliary tract. It represents the second most common primary liver cancer in the world, after hepatocellular carcinoma, and it constitutes 10–15% of hepatobiliary neoplasms and 3% of all gastrointestinal tumors. As in other types of cancers, recent studies have revealed genetic alterations underlying the establishment and progression of CCA. The most frequently involved genes are *APC*, *ARID1A*, *AXIN1*, *BAP1*, *EGFR*, *FGFRs*, *IDH1/2*, *RAS*, *SMAD4*, and *TP53*. Actionable targets include alterations of FGFRs, IDH1/2, BRAF, NTRK, and HER2. “Precision oncology” is emerging as a promising approach for CCA, and it is possible to inhibit the altered function of these genes with molecularly oriented drugs (pemigatinib, ivosidenib, vemurafenib, larotrectinib, and trastuzumab). In this review, we provide an overview of new biologic drugs (their structures, mechanisms of action, and toxicities) to treat metastatic CCA, providing readers with panoramic information on the trajectory from “old” chemotherapies to “new” target-oriented drugs.

## 1. Introduction

Cholangiocarcinoma (CCA) is a malignant neoplasm arising in the epithelium of the biliary tract. It represents the second most common primary liver cancer in the world, after hepatocellular carcinoma, and it constitutes 10–15% of hepatobiliary neoplasms and 3% of all gastrointestinal tumors [1]. In Europe, incidence rates vary between 0.4 and 1.8 per 100,000 inhabitants. The highest incidence is observed between the sixth and seventh decade of life, with a male to female ratio of 3:2 [2]. CCAs are divided into intrahepatic (iCCA) and extrahepatic (eCCA) tumors according to their anatomic location. Specifically, iCCA tumors include those arising from the proximal to the second-order bile ducts. eCCA tumors include perihilar CCA originating between the second order ducts and the insertion of the cystic duct and those arising from the epithelium distal to the insertion of the cystic duct, which are called distal CCAs [3,4]. As in other types of cancers, recent studies have revealed genetic alterations underlying the progression of CCA. The most frequently altered genes in CCA are *ARID1A*, *AXIN1*, *BAP1*, *EGFR*, *FGFRs*, *IDH1/2*, KRAS, *SMAD4*, and *TP53* (Table 1). Among the genetic alterations, there are interesting actionable targets [5]. These include the alterations of *BRAF* (p.V600E), *NTRK* fusions, and *HER2* amplifications. “Precision oncology” has recently emerged as a promising approach for CCA, and it is possible to inhibit the altered function of these genes with molecularly oriented drugs. In this review, we present an overview of the current drugs under investigation to treat metastatic CCA, providing readers with panoramic information from “old” chemotherapies to “new” target-oriented drugs.

## 2. The Chemotherapy Treatment for Advanced CCA

During the last three decades, the incidence rates of intrahepatic forms of CCA, although less common than extrahepatic ones (approximately 20% of CCAs), have increased in Western Europe and Japan compared to eastern countries [2]. From a histological point of view, over 90% of CCAs are adenocarcinomas. The remaining 10% may be mucinous, adenosquamous, squamous, clear cell, sarcomatoid, or lymphoepithelial carcinomas. Unspecific symptoms frequently generate delays in diagnosis; in fact, for only one in five patients it is possible to carry out a surgical removal with radical intent. Most patients present with locally advanced or metastatic disease primarily involving the local lymph nodes, peritoneum, and liver. Less commonly, CCA can metastasize to the lungs, bones, and brain. Biliary stenting and/or biliary bypass drainage are the most common early active symptom control (ASC) therapeutic interventions [3,4].

Specific palliative treatment options for the late stages of CCA include chemotherapy and radiotherapy. Based on the results of the randomized phase three ABC-02 study, the combination of cisplatin and gemcitabine (cisplatin 25 mg/m^2^ followed by gemcitabine 1000 mg/m^2^ on days 1 and 8 every three weeks) became the standard treatment for the first line therapy of metastatic disease. The association of these drugs achieves a response rate of 26.1% (versus 15.5%) and a median overall survival rate of 11.7 months (versus 8.1) in patients treated with gemcitabine alone (HR: 0.64; 95% CI: 0.52–0.80; *p* < 0.001) [5]. There is currently no standard second-line treatment for patients who have progressed after first-line platinum and gemcitabine. Numerous studies, largely underpowered and retrospective, have investigated the clinical efficacy of monotherapies or various chemotherapeutic combinations (irinotecan, taxanes, fluoropyrimidines, etc.), with response rates ranging from 0% to 22% [6]. A recent phase three study (ABC-06) showed a survival advantage of FOLFOX (oxaliplatin 85 mg/m^2^, folinic acid 350 mg, fluorouracil 400 mg/m^2^ bolus, and fluorouracil 2400 mg/m^2^ as a 46 h continuous intravenous infusion) as a second line over ASC in 162 patients progressing to a standard first-line therapy [7]. The median overall survival was 6.2 months (95% CI: 5.4–7.6) in the ASC plus FOLFOX group compared to 5.3 months (4.1–5.8) in the ASC group (+0.9 months; HR: 0.69; 95% CI: 0.50–0.97; *p* = 0.031). The overall survival rate in the ASC group was 35.5% (95% CI: 25.2–46.0) at 6 months and 11.4% (5.6–19.5) at 12 months compared to 50.6% (39.3–60.9) at 6 months and 25.9% (17.0–35.8) at 12 months in the ASC plus mFOLFOX group. The response rate was 5% in 81 patients in the ASC plus mFOLFOX group (one complete response and three partial responses).

Thus, we can conclude that the activity of chemotherapy in advanced CCA is questionable and, unfortunately, the prognosis remains dismal, with survival very rarely surpassing 18 months.

## 3. Targeting FGFRs in CCA

**Figure 1:**
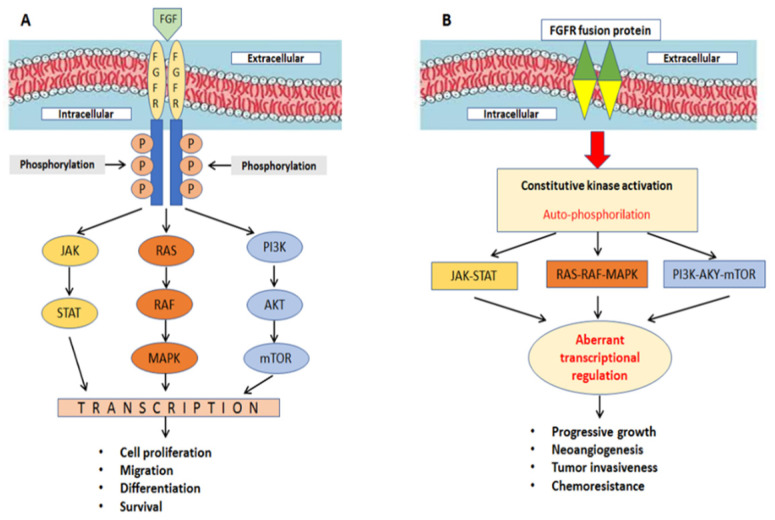
(**A**) FGFR is activated under physiologic conditions by its natural ligands. (**B**) FGF signaling can be activated by ligand-independent mechanisms.

In the last 10 years, fibroblast growth factors (FGFs) and their associated receptors (FGFRs) have been studied to exploit the therapeutic potential of inhibiting their signaling. In fact, recent studies have shown that FGFRs have important roles in the pathogenesis and biology of CCA. Indeed, in vitro data has revealed the expression of FGFRs in human CCA specimens by immunohistochemistry (FGFR1, 30% positive; FGFR2, 65% positive) [8]. The binding of FGFs is necessary for FGFR activation [9]: after ligand binding at the cell surface, FGFR dimerizes, and it activates the RAS-RAF-MAPK, PI3K-AKT-mTOR, and JAK-STAT pathways [10,11], resulting in the transcription of genes involved in cellular survival, proliferation, migration, and differentiation (Figure 1A). FGFR alterations (due to mutations, chromosomal translocations, gene fusions, or gene amplifications) lead to ligand-independent signaling activation, which, in turn, results in constitutive kinase activation causing tumor progression, neoangiogenesis, and chemoresistance (Figure 1B). In a recent study, researchers analyzed 4853 solid tumors, and among 115 CCAs, 7% harbored FGFR aberrations [12].

Fusion proteins arise from chromosomal translocations that merge two genes and their consequent protein products. *FGFR2* is the most common gene fusion in CCAs [13]. To date, hundreds of FGFR2 fusion partners have been identified, but the most common are the BICC family RNA binding protein 1 (FGFR2-BICC1), S-adenosylhomocysteine hydrolase-like protein 1 (FGFR2-AHCYL1), Periphilin 1 (FGFR2-PPHLN1), the sickle tail protein homolog (FGFR2-KIAA1217), and the coiled-coil domain containing protein 6 (FGFR2-CCDC6) [14]. In particular, among CCAs bearing FGFR2 fusions, FGFR2-BICC1 fusion was found in 28.9% of the patients [15], FGFR2-AHCYL1 in 10.6% [16], FGFR2-PPHLN1 in 16.8% [17], FGFR2-KIAA1217 in 37%, and FGFR2-CCDC6 in 1.9% [15]. Many studies have demonstrated that the oncogenic properties of FGFR2 fusion proteins can be completely suppressed by treatment with FGFR kinase inhibitors, both in vitro and in vivo [18,19,20]. FGFR-targeted treatments have entered into the therapeutic panorama of CCA patients since these agents have reported positive results in phase one/two clinical studies. In fact, recently, infigratinib [21] and pemigatinib [22] have been approved by the FDA in pretreated metastatic CCA bearing fusions or mutations of *FGFR2*. The approval for infigratinib was based on a phase two study that showed a surprising response rate (RR) of 23.1% and a median progression-free survival (PFS) rate of 7.3 months. Pemigatinib showed a robust RR of 35.5% and a median PFS rate of 6.9 months [23]. Another FGFR-targeted agent, derazantinib, was tested in CCA patients in recent years; its role was evaluated in a phase one/two, open-label trial that reported a high DCR of 82.8% and an RR of 20.7% [24]. This study included both patients with FGFR2 gene fusion and patients with FGFR2 mutations or amplifications. Finally, in recent years, futibatinib (an irreversible inhibitor) has shown activity in CCA patients pre-treated with other FGFR inhibitors, suggesting its possible role in overcoming acquired resistance [25]. Furthermore, in the FOENIX-CCA2 (NCT02052778) trial, a single-arm multicenter phase two study evaluating the activity of futibatinib in CCA patients with FGFR2 gene fusions experiencing disease progression after standard treatments (including gemcitabine plus platinum-based chemotherapy) [26] emerged an impressive RR of 34.3% in 67 of the cases enrolled.

## 4. Targeting IDH-1/2 in CCA

**Figure 2:**
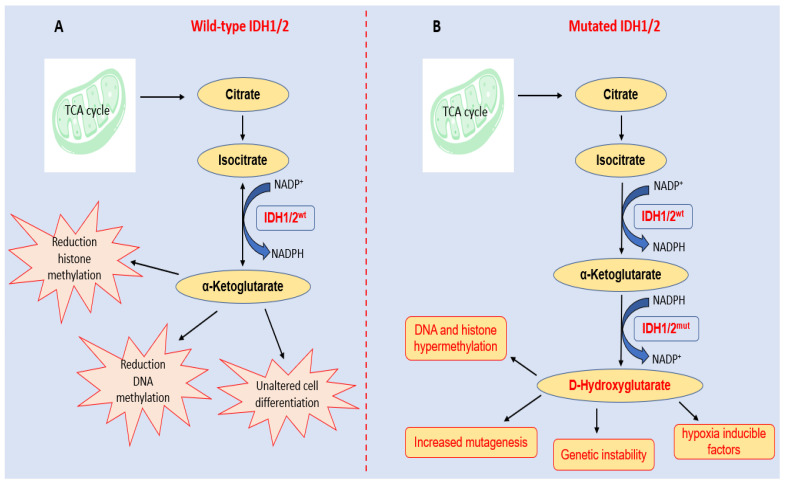
Parallel diagrams of IDH1/2′s biological roles in (**A**) normal cellular functions and (**B**) the consequence of its neomorphic activity acquired upon hot-spot mutations. (**A**) Wild-type enzymes promote normal cellular processes through metabolic pathways and the activity of α-ketoglutarate. (**B**) Mutant enzymes (IDH1/2^mut^) produce the oncometabolite D-hydroxyglutarate, a potent inhibitor of α-ketoglutarate-dependent dioxygenases, with the concomitant depletion of the NADPH, resulting in an aberrant activation of signaling pathways and sustaining cancer initiation and progression.

Isocitrate dehydrogenase 1 and 2 (IDH1 and IDH2) are cytosolic and mitochondrial enzymes that catalyse the conversion of isocitrate to α-ketoglutarate (αKG) while reducing NADP to NADPH (nicotinamide adenine dinucleotide phosphate hydrogen). NADPH is a crucial cellular reducing agent in detoxification processes involved in protection against the toxicity of reactive oxygen species and oxidative DNA damage [27]. In fact, IDH1 and IDH2 are key metabolic enzymes that frequently mutate in a variety of solid tumors, including glioma, glioblastoma, chondrosarcoma, CCA, etc. [28]. In particular, mutations in *IDH1* are detected in approximately 13% of iCCA and 1% of eCCA tumors [29]. Numerous studies have shown that IDH1/2-mutant enzymes gain neomorphic enzymatic activity (gain-of-function), converting αKG and NADPH to D-2-hydroxyglutarate (D-2HG) and NADP+ [30,31,32]. In physiological conditions, D-2HG intracellular concentration is low; in IDH1/2 mutant enzymes, high levels of D-2HG are produced. An excess of D-2HG is associated with increased histone and DNA methylation, which alters cancer cell differentiation and proliferation [33,34]. The biological roles of wild-type and mutant IGH1/2 are summarized in Figure 2.

Interestingly, high D-2HG levels cause the inhibition of hepatocellular differentiation and the uncontrolled proliferation of liver progenitor cells, suggesting that *IDH1*^mut^ may represent an early event in CCA carcinogenesis, as observed in glioblastoma and acute myeloid leukemia [35]. However, even if D-2HG produces a genetic instability contributing to mutagenesis and, consequently, cancer initiation, the accumulation of DNA damage could predispose patients to the beneficial effects of radiotherapy, chemotherapy, and immunotherapy [36].

*IDH1* mutations are more common than those of *IDH2* [37]. After the crucial discovery of gain-of-function properties in the IDH1 mutated enzyme, research has focused on the development of small synthetic molecules able to inhibit the aberrant activity of *IDH1*^mut^. Inhibitors of *IDH1*^mut^ (IDH305 and FT-2102), *IDH2*^mut^ (AG221), and pan-*IDH1/2*^mut^ (AG881) are under investigation for CCA patients [38,39,40,41]. All these molecules demonstrate rapid oral absorption, slow elimination rates, and long half-lives. In particular, in *IDH1*-mutated CCA patients’ refractories to previous systemic therapies, ivosidenib (AG120) showed a significant—albeit small—improvement in the median PFS versus the placebo (2.7 months versus 1.4 months; HR: 0.37; 95% CI: 0.25–0.54; *p* = 0.001) and a DCR of 53.2% (3 RP and 63 SD out of 124 patients). Based on the results of this randomized phase three study, the FDA approved the use of ivosidenib in this clinical setting [22].

## 5. Inhibition of *BRAF* p.V600E in CCA

**Figure 3:**
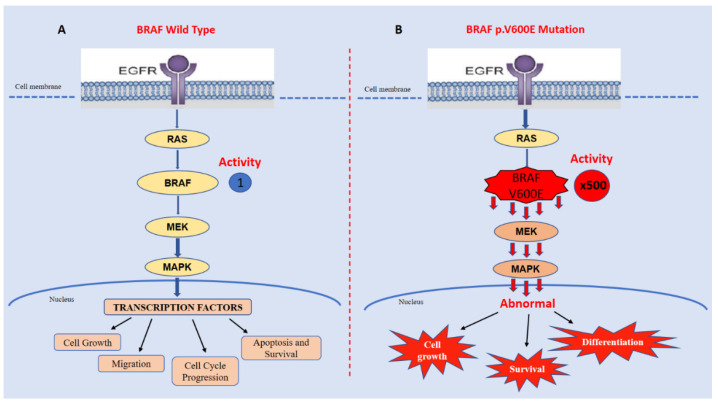
The p.V600E-mutated BRAF has approximately 500-fold greater activity than its wild-type form (**B** vs. **A**), resulting in excessive MAPK signaling and abnormal cell growth, survival, and differentiation.

The mitogen-activated protein kinase (MAPK) pathway is involved in the crucial cellular processes of proliferation and survival [42]. BRAF is a serine/threonine protein kinase representing an oncogenic driver in many human cancers [43]. It is an important player in the EGFR (epidermal growth factor receptor)-mediated MAPK (mitogen-activated protein kinase) pathway via activation through the RAS small GTPase [44]. BRAF is crucial for activating the MAPK pathway, profoundly influencing cell growth, proliferation, and differentiation, and it is involved in several cellular processes, including cell migration, apoptosis, and survival [45]. In particular, mutations at codon 600 result in the constitutive activation of BRAF and aberrant MAPK signaling (Figure 3).

*BRAF* mutations have been found in several malignancies including melanoma, non-small cell lung cancer, CCA, and colorectal cancer [43,46,47,48]. To date, more than 50 *BRAF* mutations have been identified in CCA, but the most common is the p.V600E variant [49]. In an interesting study, *BRAF* p.V600E mutation detected by immune-histochemistry in CCAs was associated with (i) a higher TNM stage, (ii) resistance to systemic chemotherapy, and (iii) an aggressive clinical course with worse survival rates (median survival 13.5 months versus 37.3 in wild-type patients) [50]. The first attempt at evaluating the possibility of targeting *BRAF* mutations in metastatic biliary tract cancers was a phase two basket trial [51]. In this study, a dismal response rate of 12% (one partial response/eight total patients) was reported with vemurafenib, likely related to the high burden of disease and poor patient conditions. Later, successful combination therapies involving BRAF inhibitors (i.e., vemurafenib or dabrafenib) for the treatment of metastatic CCAs were reported [52,53]. In most cancers bearing *BRAF* mutations, patients treated with BRAF inhibitors develop disease progression within a few months from the start of treatment. It has been demonstrated that resistance is predominantly mediated by downstream MAPK pathway alterations, including *MEK* activating mutations. For this reason, the concomitant BRAF and MEK inhibition overcomes the acquired resistance to BRAF inhibitors and potentiates the anti-tumor effects [54,55]. Of note, the interesting phase two study by Subbiah and colleagues demonstrated a promising efficacy of dabrafenib (a BRAF inhibitor) and trametinib (a MEK inhibitor) in *BRAF* p.V600E-mutated biliary tract cancers, with an ORR of 51% and a median PFS and median OS of 9.0 months and 14.0 months, respectively [56]. To date, the last is still considered the best anti-tumor approach in *BRAF*^mut^ CCAs [52,57]. The clinical benefit highlighted with this drug combination represents an important step forward in the management of this group of tumors. We believe that, given the relevant frequency of the *BRAF* p.V600E mutation in CCAs, genetic testing should always be performed in these neoplasms.

## 6. *NTRK* Fusions: Role in CCAs

The neurotrophic tyrosine receptor kinase (*NTRK-1*, -*2*, -*3*) genes encode for TRK-A, -B, and -C (Tropomyosin receptor kinase-A). These are the high affinity receptors for the “neurotrophin” (or nerve growth factor, NGF) that is crucially involved in neural development. NTRK is a member of the tyrosine kinases family associated with the MAPK signaling pathway [58]. Each protein consists of an intracellular kinase domain, an extracellular ligand-binding domain, and a transmembrane region [59]. Upon activation, they strongly stimulate cell differentiation, proliferation, and survival. Fusions or genomic translocations in NTRK genes determine a constitutive activation of the associated receptor tyrosine kinases. The products of these aberrant fusions have been found as key-driver alterations in many different malignant tumors, including CCAs [60]. For this reason, NTRK fusion inhibitors have been considered a “tumour-agnostic” treatment in NTRK fusion-positive cancers. Notably, TRKs are constitutively activated by various mechanisms in malignancies, but the most frequent is represented by the *NTRK* gene fusions.

**Figure 4:**
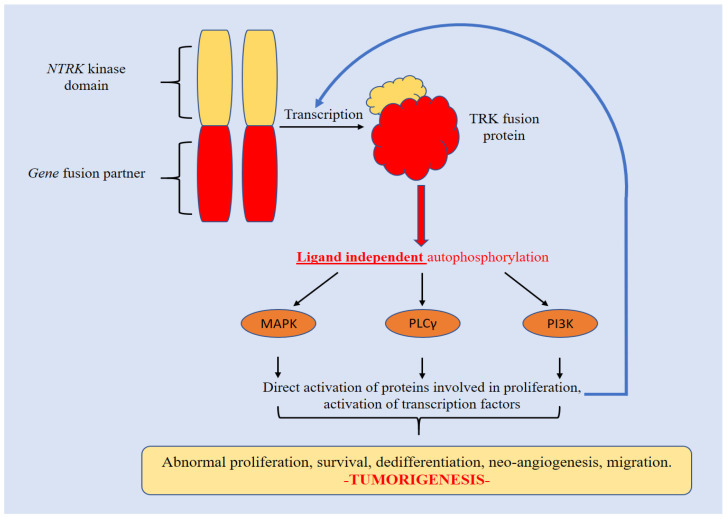
The fusion product is functionally aberrant. Auto-phosphorylation occurs independently from ligands, causing a strong activation of TRK activity that, in turn, triggers the transduction of the downstream signaling pathways (MAPK-PLCγ-PI3K) involved in tumorigenesis.

In the literature, approximately 80 fusion partners have been described. In this mechanism, the 3′ region of the *NTRK* gene is rearranged, intra- or inter-chromosomally, and it connects with the 5′ sequence of the fusion partner gene [61]. The fusion eliminates the ligand binding site, resulting in ligand-independent dimerization and a phosphorylation cascade that potently triggers the proliferation and growth of cancer cells [62]. A schematic representation of the molecular cascade sustained by *NTRK* fusions is provided in Figure 4.

However, the estimated prevalence of NTRK fusions in CCAs is very low, and they are considered rare events (0.75%) [63]. Furthermore, even if gene fusions may potentially involve all *NTRK* genes (1–4), in CCAs only *NTRK-1* has been detected so far [59]. The *NTRK* gene, fusion partner, and chromosomal localization involved in CCAs [64,65,66,67] are summarized in Table 2.

Over the past decade, *NTRK* fusions have been intensively study as potential antitumor targets focusing on the development of a large number of TRK small molecule inhibitors. There are only two NTRK inhibitors that have been approved by both the FDA and the EMA: larotrectinib and entrectinib. These inhibitors have achieved high response rates and durable responses in metastatic solid tumors (including those of patients with CCA). Larotrectinib and entrectinib were associated with ORRs of 75% and 57%, respectively. The median response duration was 10 months with larotrectinib, though none was reached with entrectinib [68,69]. These high response rates accounted for the pharmaceutical authorities’ approvals for their use in patients with NTRK-fusion-positive cancers refractories to standard treatments. Interestingly, the NCCN guidelines currently recommend NTRK inhibitors as first or subsequent treatment lines of therapy in *NTRK*-fusion-positive CCAs.

## 7. *HER2* Amplification in CCA

The human epidermal growth factor receptor 2 (EGFR2 or HER2 or ERBB2) belongs to a family of tyrosine kinase receptors with four distinct domains that, following ligand binding, undergo to homo- or hetero-dimerization [70]. Consequently, the intrinsic tyrosine kinase domain activates and triggers a downstream signaling cascade, including the MAPK and PI3K/PKB pathways, which are essential for cell growth/proliferation and malignant transformation [71]. Currently, these represent solid predictive biomarkers for targeted-therapy in gastric, oesophageal, and breast cancers [72,73]. However, recent research has demonstrated *HER2* aberrations in CCAs.

In an interesting and large study [74], *HER2* amplification was found in 1.4% of more than 400 surgically resected and histologically confirmed CCAs. More variable results have been reported in South American or Asian populations [75,76,77,78,79] (from 0.1% to 20%). These differences are likely attributable to the remarkable inconsistency in HER2 testing methods used so far. However, based on these data, we cannot rule out that HER2 could also be a significant prognosticator in CCAs. Interestingly, some studies, although downsized (case reports or small retrospective series), have reported partial responses in patients with HER2-positive CCAs receiving anti-HER2-targeted therapy [80,81,82]. In the future, prospective, comparative, and large trials are needed to confirm the efficacy of anti-HER2 in the management of CCAs. However, one of the most promising molecules to target amplified *HER2* in CCA is trastuzumab; it is a humanized monoclonal antibody able to bind the extracellular binding domain of HER2. It suppresses the signaling pathways and induces HER2 degradation [83]. Currently, trastuzumab is under evaluation in a phase one study in combination with the use of tipifarnib (a farnesyltransferase inhibitor known to block RAS signaling) [84]. Trastuzumab as an antibody-drug conjugate with deruxtecan (DS-8201) is also being evaluated in CCA patients with HER2 alterations [85]. Neratinib, an oral, irreversible pan-HER tyrosine kinase inhibitor, is under investigation in the phase two SUMMIT basket trial study (NCT01953926); encouraging results, with an objective response rate of 16% and a clinical benefit rate of 28% in 25 patients with CCA, have been obtained [86].

## 8. Overview of Target-Oriented Drugs in CCA

**Figure 5:**
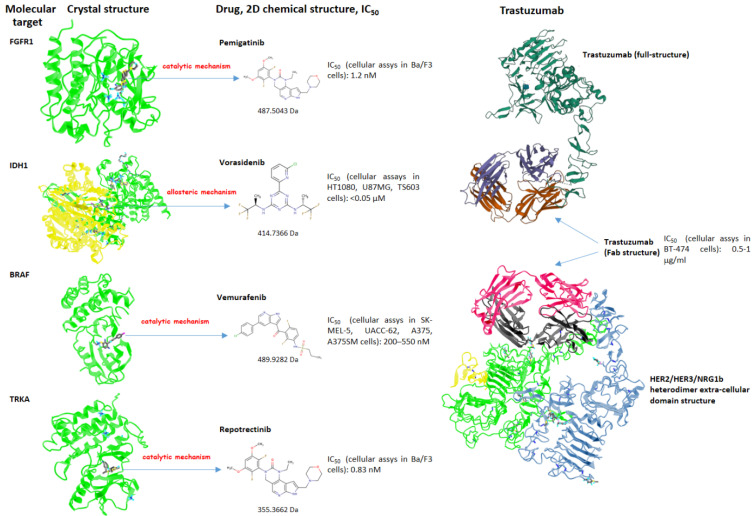
Schematic representation of the molecular targets and their crystal structures, inhibitory small molecules with their 2D chemical structures, molecular weights, and IC_50_ (for each IC_50,_ the cellular model used to establish it is reported). For exploring the single amino acids involved in the structural interactions between the chemical compounds and the molecular target in a 3D navigation perspective, please visit the online research tool of the NCBI (National Center for Biotechnology Information) [87]. When the crystal structure of the protein in the complex with the molecule reported in the article was not available, we reported the structure bounded to a chemical compound belonging to the same functional class (vorasidenib for ivosidenib and repotrectinib for larotrectinib, both interacting with the same functional regions of IDH1 and TRKA, respectively). The inhibitory effect through the “allosteric mechanism” signifies that the molecule inhibits the target by binding to an allosteric site that is distant from the catalytic/active site of the kinase. In this case, a conformational change is induced, preventing access to the enzymatic pocket.

An overview of the pharmacodynamics and molecular characteristics of the target-oriented drugs used in the treatment of CCA is provided in Table 3 and Figure 5. Their toxicities are manageable and profoundly different from those registered with chemotherapy. These drugs are emerging examples of “precision oncology” based on the specific genetic profile of CCAs. Ideally, advances in the knowledge of the role of still-not-actionable genetic alterations (Table 1) and of the mechanism of resistance will lead to the exploration of combined, sequential treatments and re-challenges in the future.

## 9. Conclusions and Perspectives

CCA incidence is increasing and, unfortunately, its prognosis remains very poor. The reasons are large genetic heterogeneity (from a molecular point of view) and scarce chemotherapy responsiveness (from a clinical point of view). These characteristics render the CCA a perfect model to study and apply innovative and molecularly oriented drugs. A new class of drugs is now emerging, and its application is necessarily preceded by the genetic profiling of CCA towards a “precision oncology” (i.e., next-generation sequencing). Every patient will deserve a “from bench to bad” approach and commitment. In this expanding context, future clinical research paradigms (first-line biologic therapies, repeated genetic assessments with liquid biopsy, associations, biologics re-challenges, etc.) must be pursued that revolutionize our dogmatic approaches to sequential treatments, molecular testing, and response monitoring.

## Figures and Tables

**Table 1:** Genetic landscape of cholangiocarcinoma.

Acronym	Full Name	Incidence (%)
APC	Adenomatous polyposis coli	15
ARID1A	AT-rich interaction domain 1A	25
AKT1	Homologue retrovirus kinase isolated from AKT-8(-1)	<5
AXIN1	Axis inhibition protein 1	40
BAP1	BRCA1-associated protein 1	25
BRAF	v-raf murine sarcoma viral oncogene homolog B1	20
BRCA1	Breast Cancer gene 1	<1
BRCA2	Breast Cancer gene 2	3
CDKN2A/B	Cyclin-dependent kinase inhibitor 2A/B	15
CTNNB1	Catenin Beta 1	8
c-MET	Cellular-mesenchymal epithelial transition factor	<5
EGFR	Epidermal growth factor receptor	20
FGFR2	Fibroblast growth factor receptor 2	15
HER2	Human epidermal growth factor receptor 2	<10
IDH1/2	Isocitrate dehydrogenase 1/2	15
KRAS	Kirsten rat sarcoma viral oncogene homolog	20
MEK	Mapk/erk kinase	15
NTRK	Neurotrophic tyrosine receptor kinase	<1
PIK3CA	Phosphatidylinotitol 3-kinase catalytic subunit alpha	15
ROS1	ROS proto-oncogene 1	10
SMAD4	Mothers against decapentaplegic homolog 4	25
TP53	Tumor protein 53	35
VEGF	Vascular endothelial growth factor	15

**Table 2:** NTRK gene fusions identified in CCAs.

Gene/Fusion Partner	Chromosomal Localization	Ref.
NTRK1/LMNA (Lamin A)	1q22	[64,67]
NTRK1/TPM3 (Tropomyosin 3)	1q21.3	[64]
NTRK1/RABGAP1L (RAB GTPase Activating Protein 1 Like)	1q25.1	[65,67]
NTRK1/PLEKHA6 (Pleckstrin Homology Domain Containing A6)	1q32.1	[66]

**Table 3:** Molecular and clinical characteristics of the target-based drugs used in CCA.

Target	Drug	Molecular Weight (Da)	Type of Inhibition	IC_50_ *	Most Common Clinical Toxicities (All Grades)
FGFR	Pemigatinib	487.50	Reversible	0.5 nM/L *	Diarrhea, fatigue, alopecia, and eye tox
	Infigratinib	560.48	Reversible	1.4 nM/L *	Eye tox, stomatitis, and fatigue
	Derazantinib	468.57	Reversible	1.8 nM/L *	Eye tox, stomatitis, and fatigue
	Futibatinib	418.45	Irreversible	1.4 nM/L *	Nail tox, fatigue, and musculoskeletal tox
IDH1	Ivosidenib	582.96	Reversible	70.0 nM/L	Diarrhea, neutropenia, leukocytosis, and fatigue
BRAF	Vemurafenib	482.92	Reversible	31 nM/L	Alopecia, arthralgia, fatigue, and eye tox
	Dabrafenib	519.56	Reversible	0.7 nM/L	Fever, neutropenia, and fatigue
NTRK	Larotrectinib	526.51	Reversible	11 nM/L **	Anemia, fatigue, and nausea
	Entrectinib	560.63	Reversible	1 nM/L **	Dysgeusia, fatigue, and diarrhea
HER2	Trastuzumab	145,531.5	Reversible	1 mg/mL	Fever, nausea, allergy, and diarrhea

Da: Daltons; IC50: inhibitory concentration by 50%; Tox: toxicity. * All IC50, except for HER row, refer to cell-free assays (kinase activity); * IC50 on FGFR2 activity; ** IC50 on TRKA activity.

## Data Availability

Not applicable.
